# Editorial: Experts' opinion in medicine 2022

**DOI:** 10.3389/fmed.2023.1296196

**Published:** 2023-10-10

**Authors:** Victoria I. Bunik

**Affiliations:** ^1^Belosersky Institute of Physicochemical Biology, Lomonosov Moscow State University, Moscow, Russia; ^2^Faculty of Bioengineering and Bioinformatics, Lomonosov Moscow State University, Moscow, Russia; ^3^Department of Biochemistry, I.M. Sechenov First Moscow State Medical University, Moscow, Russia

**Keywords:** thiamine (B1), glutamine (GLN) metabolism, amino acid deprivation, lysine for protein glutarylation, gamma-amidase, benfotiamine in Alzheimers disease, methionine salvage pathway, AI and primary care

Introducing personalized therapies requires a better, than implemented currently, coupling of basic research to medicine. The current topic is dedicated to promote this coupling through presenting the basic research that has clear medical implications. The medicine-inspired studies of neurodegenerative diseases (Gibson et al.) and cancer (Cooper et al.; Pokrovsky et al.) demonstrate how the knowledge on molecular mechanisms may improve diagnostics and treatments of human diseases. To support the on-going discussion on artificial-intelligence-based approaches to solve medical challenges, we also include an opinion reflecting on possible advantages and drawbacks of such approaches (Mainous III).

Gibson et al. discuss results of a pilot clinical trial of benfotiamine (a pharmacological form of thiamine, or vitamin B1) in patients with Alzheimer's disease, regarded in view of impressive amount of data on the role of thiamine in the nervous system. Pharmacological doses of thiamine, i.e. the doses much higher than naturally occurring ones, are required for the positive action of thiamine in the age-associated neurodegenerative diseases. This is most probably due to a multitude of the age-associated factors and conditions altering the thiamine delivery to tissues. As multiple drugs including widely used antidiabetics metformin, compete with thiamine for cellular thiamine transporters (1), individual differences in thiamine transporters caused by genetic or epigenetic factors, may increase interpersonal variations in the brain thiamine pool and its response to the administration of thiamine and derivatives. Besides, perturbed thiamine phosphorylation, such as an imbalance in the phosphorylation and dehosphorylation of blood thiamine, is known in the patients with Alzheimer's disease vs. the control group (2). Hence, the impaired cellular transport and/or metabolism of thiamine may be compensated by unnaturally high levels of administered thiamine and derivatization into the membrane penetrating forms. In the pilot study, benfotiamine reduces cognitive decline (3) and decreases serum indicators of Alzheimer's disease (4), when the drug is supplemented at a mild stage. The results support the hypothesis that perturbed metabolism of thiamine is an essential factor in Alzheimer's disease and may be a therapeutic target.

The most obvious pathophysiological mechanism of thiamine deficiency is a decrease in glucose metabolism due to insufficient coenzyme form of thiamine, thiamine diphosphate (ThDP). Nevertheless, in Alzheimer's disease patients, the blood activity of thiamine diphosphatase, the enzyme decreasing the levels of the coenzyme ThDP, does not correlate with the fasting glucose levels as strong as the blood activity of thiamine monophosphatase, the enzyme increasing the levels of thiamine *mono*phosphate, does (2). Hence, in addition to the essential coenzyme role, a less appreciated non-coenzyme action of thiamine and its non-coenzyme derivatives transported across biological membranes, deserves attention. For example, the binding of ThDP to the master regulator of glucose metabolism, p53, is competitive to that of DNA, resulting in the regulation of p53-dependent transcriptional activity by the coenzyme (5). It has also been long known that, apart from the thiamine coenzyme pool, there is a rapidly metabolized pool of thiamine and/or its natural derivatives, participating in neuronal excitation (6). Including thiamine *tri*phosphate, the rapidly metabolized pool is involved in the acetylcholine action at synapses, where thiamine co-releases with acetylcholine (7, 8), and a non-coenzyme derivative thiamine *tri*phosphate phosphorylates rapsyn (9). Such highly dynamic and local processes may involve only a minor part of the total brain thiamine pool and/or rely on the time-dependent thiamine gradients across membranes. Nevertheless, their utmost importance for the brain function may underly multiple observations that the positive action of thiamine in nervous system may occur without significant increases in the brain levels of the coenzyme ThDP. Gibson et al. draw our attention to this underestimated complexity of the brain action of thiamine.

The opinions of Cooper et al. and Pokrovsky et al. consider molecular mechanisms of plasticity of cancer metabolism of amino acids. The more we know about the metabolic differences between the cancer and normal cells, the more cancer-specific or cancer-heightened processes may be employed to fight malformations without significant side effects. For instance, addiction of cancer cells to glutamine provides such a long-known anticancer target as glutaminase. Cooper et al. consider a much less known and underestimated alternative to the glutaminase pathway, where glutamine generates α-ketoglutarate through the ω-amidase pathway. Inhibition of ω-amidase may have minimal effects on normal tissues, but be deleterious to the glutamine-addicted tumors, especially those with elevated expression of the enzyme. Existence of this alternative to the glutaminase pathway may be a reason why the glutaminase inhibitors have not yet resulted in therapeutic applications (10). By inducing the alternative ω-amidase pathway, cancer cells may well acquire resistance to the glutaminase-directed drugs. Thus, combinatorial therapies blocking both the glutaminase and ω-amidase pathways may be more successful to kill the glutamine-addicted cancer cells, than the impairment of a single pathway by glutaminase inhibitors.

Apart from blocking the enzymes, essential for the amino acid metabolism in cancer cells, one may also deprive cancer cells of essential amino acids. These anticancer approaches and underlying molecular mechanisms are reviewed by Pokrovsky et al.. Several forms of the FDA-approved bacterial asparagine-degrading enzyme asparaginase are currently available for the treatment of acute lymphoblastic leukemia. The ongoing studies also focus on the depletion of L-arginine, L-methionine and L-lysine. Overall, the research has shown that such therapeutic approaches are efficient in the cancer cells that lack the enzymes replenishing the depleted amino acid. Hence, identifying molecular mechanisms of the plasticity of cancer cell metabolism and characterizing expression of the tumor enzymes before, during and after exposure to the amino acid deprivation therapies, is of diagnostic importance (Pokrovsky et al.).

Apart from the function of all proteinogenic amino acids as the protein building blocks, that causes high sensitivity to amino acid deprivation in strongly proliferating cells, many amino acids have additional specific roles as participants of different biosynthetic and signaling processes. Sensitivity to deprivation of these amino acids is further elevated, depending on the cancer-cell-specific metabolism. For instance, glutamine is used by cancer cells to generate essential metabolite α-ketoglutarate under hypoxic conditions in the pathways considered above, and as a nitrogen source for biosynthesis of amino acids, nucleotides and antioxidant glutathione. Lysine supports ketogenesis that is activated in some cancer cells to overcome drug action (11). Lysine degradation through the *DHTKD1*-encoded α-ketoadipate dehydrogenase produces glutaryl-CoA for glutarylation of proteins, including those essential for malignant transformation, such as histones (12) and pyruvate dehydrogenase complex (13). Methionine is required for DNA methylation and for initiating the protein and polyamine synthesis. Remarkably, the α-keto analog of methionine, α-keto-γ-methiolbutyrate, generated in the methionine salvage pathway, is an excellent α-keto substrate for the glutamine transamination in the ω-amidase pathway (Cooper et al.). Hence, blockade of ω-amidase working in tandem with glutamine transaminase, would not only prevent the production of α-ketoglutarate from glutamine through the ω-amidase pathway, but also interfere with the methionine regeneration. Enzymes of the methionine salvage pathway producing α-keto-γ-methiolbutyrate are targets for anticancer therapies (14). In this regard, expression of the methionine salvage pathway enzymes linked to ω-amidase, may provide important predictors for anticancer effect of methionine depletion, additional to those considered by Pokrovsky et al..

Thus, basic research on (i) cancer-specific features of the amino acids metabolism, (ii) pathways activated to overcome the amino acid deficiency, and (iii) molecular mechanisms of the cancer cell dependence on specific amino acids, helps to define new anticancer targets, combinatorial therapies and personalized markers to predict the therapies efficacies.

Artificial-intelligence-based approaches are supposed to promote the translation of basic research to medicine by providing a better understanding of the disease- or drug-affected pathways (15, 16), particularly those assessed by modern imaging techniques (17–20). Artificial intelligence may also help patients to learn more about their diseases and the needed control measures. However, one must carefully consider the ways to use the artificial intelligence in the organization and function of health system. Artificial intelligence should not disturb the vital interaction between the doctors and patients. Using an example of Luddites, the expert opinion of Mainous III shows that inspirations and progress brought about by new technologies, do not exclude their inappropriate usage. To benefit the well-being of patients, application of the artificial-intelligence-based approaches must help the primary care doctors, but not substitute the doctors. Although the substitution would be cost-effective to benefit insurance companies and corporations, disappearance of the primary care doctors is a disadvantage for patients and society. While earlier the doctors could “sniff” the ketone bodies to diagnose the disease, the current trend is not to provide an expert opinion without prescribing a patient to go through a number of tests. However, what if the tests are not available or the insurance is not appropriate? Elimination of the human expertise in medical examination by analytical tests prescribed through online diagnostics by artificial intelligence will cause unnecessary delays, negatively affecting the time of the first aid. Besides, taken over by the universal formalized approaches of artificial intelligence lacking non-verbal components, the overall accumulated knowledge on personal medical examination will undergo a decay. As a result, Mainous III concludes that “the use of virtual-first primary care without a physical exam… is an open question” with the answer far from obvious.

## Author contributions

VB: Conceptualization, Project administration, Writing—original draft, Writing—review and editing.

## References

[1] Aleshin VA Mkrtchyan GV Bunik VI. Mechanisms of non-coenzyme action of thiamine: protein targets and medical significance. Biochemistry (Mosc). (2019) 84:829–50. 10.1134/S0006297919080017 31522667

[2] Pan X Sang S Fei G Jin L Liu H Wang Z. Enhanced activities of blood thiamine diphosphatase and monophosphatase in Alzheimer's disease. PLoS ONE. (2017) 12:e0167273. 10.1371/journal.pone.0167273 28060825 PMC5218390

[3] Gibson GE Luchsinger JA Cirio R Chen H Franchino-Elder J Hirsch JA. Benfotiamine and cognitive decline in Alzheimer's disease: results of a randomized placebo-controlled phase iia clinical trial. J Alzheimers Dis. (2020) 78:989–1010. 10.3233/JAD-200896 33074237 PMC7880246

[4] Bhawal R Fu Q Anderson ET Gibson GE Zhang S. Serum metabolomic and lipidomic profiling reveals novel biomarkers of efficacy for benfotiamine in Alzheimer's disease. Int J Mol Sci. (2021) 22:13188. 10.3390/ijms222413188 34947984 PMC8709126

[5] McLure KG Takagi M Kastan MB. Nad+ modulates P53 DNA binding specificity and function. Mol Cell Biol. (2004) 24:9958–67. 10.1128/MCB.24.22.9958-9967.2004 15509798 PMC525472

[6] Parkhomenko YM Pavlova AS Mezhenskaya OA. Mechanisms responsible for the high sensitivity of neural cells to vitamin B1 deficiency. Neurophysiology. (2016) 48:429–48. 10.1007/s11062-017-9620-3

[7] Minz B. Cocarboxylase and the synthesis of acetylcholine. Proc Soc Exp Biol Med. (1946) 63:280. 10.3181/00379727-63-15573P 20277722

[8] Von Muralt A. The role of thiamine (Vitamin B1) in nervous excitation. Exp Cell Res. (1958) 14:72–9. 13586284

[9] Nghiem HO Bettendorff L Changeux JP. Specific phosphorylation of torpedo 43k rapsyn by endogenous kinase(s) with thiamine triphosphate as the phosphate donor. FASEB J. (2000) 14:543–54. 10.1096/fasebj.14.3.543 10698970

[10] Wang D Li X Gong G Lu Y Guo Z Chen R. An updated patent review of glutaminase inhibitors (2019-2022). Expert Opin Ther Pat. (2023) 33:17–28. 10.1080/13543776.2023.2173573 36698323

[11] Palma FR Ratti BA Paviani V Coelho DR Miguel R Danes JM. AMPK-deficiency forces metformin-challenged cancer cells to switch from carbohydrate metabolism to ketogenesis to support energy metabolism. Oncogene. (2021) 40:5455–67. 10.1038/s41388-021-01943-x 34290400 PMC8669831

[12] Xu Y Shi Z Bao L. An expanding repertoire of protein acylations. Mol Cell Proteomics. (2022) 21:100193. 10.1016/j.mcpro.2022.100193 34999219 PMC8933697

[13] Boyko AI Karlina IS Zavileyskiy LG Aleshin VA Artiukhov AV Kaehne T. Delayed impact of 2-oxoadipate dehydrogenase inhibition on the rat brain metabolism is linked to protein glutarylation. Front Med (Lausanne). (2022) 9:896263. 10.3389/fmed.2022.896263 35721081 PMC9198357

[14] Cooper AJL Dorai T Pinto JT Denton TT. Metabolic Heterogeneity, Plasticity, and Adaptation to “Glutamine Addiction” in Cancer Cells: The Role of Glutaminase and the Gtomegaa [Glutamine Transaminase-Omega-Amidase (Glutaminase Ii)] Pathway. Biology. (2023) 12:1131. 10.3390/biology12081131 37627015 PMC10452834

[15] Hashemi N Hao B Ignatov M Paschalidis IC Vakili P Vajda S. Improved Prediction of Mhc-Peptide Binding Using Protein Language Models. Front Bioinform. (2023) 3:1207380. 10.3389/fbinf.2023.1207380 37663788 PMC10469926

[16] Wang L Sun C Xu X Li J Zhang W. A neighborhood-regularization method leveraging multiview data for predicting the frequency of drug-side effects. Bioinformatics. (2023) 39:btad532. 10.1093/bioinformatics/btad532 37647657 PMC10491955

[17] Huynh BN Groendahl AR Tomic O Liland KH Knudtsen IS Hoebers F. Head and neck cancer treatment outcome prediction: a comparison between machine learning with conventional radiomics features and deep learning radiomics. Front Med. (2023) 10:1217037. 10.3389/fmed.2023.1217037 37711738 PMC10498924

[18] Gottlich HC KP Gregory AV. Kline TL. AI in the loop: functionalizing fold performance disagreement to monitor automated medical image segmentation workflows. Front Med. (2023) 3:1223294. 10.3389/fradi.2023.1223294 37780641 PMC10540615

[19] Piansaddhayanon C Koracharkornradt C Laosaengpha N Tao Q Ingrungruanglert P Israsena N. Label-free tumor cells classification using deep learning and high-content imaging. Sci Data. (2023) 10:570. 10.1038/s41597-023-02482-8 37634014 PMC10460430

[20] Rouhollahi A Willi JN Haltmeier S Mehrtash A Straughan R Javadikasgari H. Cardiovision: a fully automated deep learning package for medical image segmentation and reconstruction generating digital twins for patients with aortic stenosis. Comput Med Imaging Graph. (2023) 109:102289. 10.1016/j.compmedimag.2023.102289 37633032 PMC10599298

